# Cortical Excitability Measures May Predict Clinical Response to Fampridine in Patients with Multiple Sclerosis and Gait Impairment

**DOI:** 10.3390/brainsci9120357

**Published:** 2019-12-05

**Authors:** Rechdi Ahdab, Madiha M. Shatila, Abed Rahman Shatila, George Khazen, Joumana Freiha, Maher Salem, Karim Makhoul, Rody El Nawar, Shaza El Nemr, Samar S. Ayache, Naji Riachi

**Affiliations:** 1 Lebanese American University Gilbert and Rose Mary Chagoury School of Medicine, Byblos P.O. Box 36, Lebanon; chadahdab@gmail.com (R.A.); mado.shatila@gmail.com (M.M.S.); gkhazen@lau.edu.lb (G.K.); joumanafreiha@gmail.com (J.F.); Maher.Salem@lau.edu (M.S.); karim.makhoul@lau.edu (K.M.); roudynawar@hotmail.com (R.E.N.); shaza.el-nemr@hotmail.com (S.E.N.); 2 Hamidy Medical Center, Tripoli 1300, Lebanon; 3 Neurology Division, Lebanese American University Medical Center Rizk Hospital, Beirut P.O Box 11-3288, Lebanon; 4 Makassed General hospital, Beirut P.O. Box 11-6301, Lebanon; ashatila1@hotmail.com; 5 Computer Science and Mathematics Department, Lebanese American University, Byblos P.O. Box 36 , Lebanon; 6 Service de Physiologie-Explorations Fonctionnelles, Hôpital Henri Mondor, Assistance Publique—Hôpitaux de Paris, 51 avenue de Lattre de Tassigny, 94010 Créteil, France; samarayache@gmail.com; 7 EA 4391, Excitabilité Nerveuse et Thérapeutique, Université Paris-Est-Créteil, 94010 Créteil, France

**Keywords:** short intracortical inhibition, intracortical facilitation, fampridine, multiple sclerosis, walking disability

## Abstract

*Background:* Most multiple sclerosis (MS) patients will develop walking limitations during the disease. Sustained-release oral fampridine is the only approved drug that will improve gait in a subset of MS patients. *Objectives:* (1) Evaluate fampridine cortical excitability effect in MS patients with gait disability. (2) Investigate whether cortical excitability changes can predict the therapeutic response to fampridine. *Method:* This prospective observational study enrolled 20 adult patients with MS and gait impairment planned to receive fampridine 10 mg twice daily for two consecutive weeks. Exclusion criteria included: Recent relapse (<3 months), modification of disease modifying drugs (<6 months), or Expanded Disability Status Scale (EDSS) score >7. Neurological examination, timed 25-foot walk test (T25wt), EDSS, and cortical excitability studies were performed upon inclusion and 14 days after initiation of fampridine. *Results:* After treatment, the mean improvement of T25wt (ΔT25wt) was 4.9 s. Significant enhancement of intra-cortical facilitation was observed (139% versus 241%, *p* = 0.01) following treatment. A positive correlation was found between baseline resting motor threshold (rMT) and both EDSS (*r* = 0.57; *p* < 0.01) and ΔT25wt (*r* = 0.57, *p* = 0.01). rMT above 52% of the maximal stimulator output was found to be a good predictor of a favorable response to fampridine (accuracy: 75%). *Discussion:* Fampridine was found to have a significant modulatory effect on the cerebral cortex, demonstrated by an increase in excitatory intracortical processes as unveiled by paired-pulse transcranial magnetic stimulation. rMT could be useful in selecting patients likely to experience a favorable response to fampridine.

## 1. Introduction

Most patients with multiple sclerosis (MS) will develop walking limitations at some point in the course of the disease [1]. The impact of such limitations on daily activities and quality of life is substantial [2]. Currently, sustained-release oral fampridine is the only approved drug for the symptomatic treatment of walking disability due to MS. Fampridine (4-aminopyridine) is a voltage-dependent potassium channel-blocker that restores action potential conduction in poorly myelinated central nerve fibers, with a positive impact on synaptic transmission and neuronal excitability [3,4]. Unfortunately, only a subset of patients with MS and walking disabilities are responders to fampridine [5]. Limited data suggest that more advanced walking disability [6,7] and prolonged central conduction times [6,8] are predictive of a favorable response to treatment. The predictive value of more advanced measurements of cortical dysfunction have not been studied. 

Changes in cortical excitability are frequent in MS, especially in more advanced stages of the disease [9,10,11,12]. Such changes are influenced by several factors, including the clinical form, stage of the disease, and degree of disability. It has been suggested that some excitability parameters correlate with the level of disability. This concerns more particularly the short-interval intracortical inhibition (SICI), intracortical facilitation (ICF), and resting motor threshold (rMT) [9,10,11,12]. 

This study was designed to evaluate the effect of slow-release fampridine on cortical excitability in patients with MS and gait disabilities. In addition, we set out to investigate whether cortical excitability changes could serve as predictors of therapeutic response to fampridine.

## 2. Materials and Methods

### 2.1. Study Design

This prospective observational study included adult patients with MS and gait impairment. All patients planned to receive fampridine in the period between April 2016 and August 2017 were asked if they were willing to participate in the study. If so, the protocol was thoroughly explained and written informed consent was obtained. The study was approved by our institutional review board. 

### 2.2. Subjects

Eligible patients were adults (aged 18 and above) with a definite diagnosis of MS according to the 2010 McDonald criteria [13]. All patients had gait disturbance and were planned to receive a 2-week fampridine trial to assess the clinical impact of the drug on walking speed. This is an essential step needed to secure approval for the treatment from third party payers in our country. Patients were required to have recordable motor evoked potentials (MEPs) in at least one hand. Those with a recent relapse (<3 months), a recent modification of disease modifying drugs (prior 6 months), or an EDSS above 7 were excluded. Patients with contraindication to fampridine (seizures, pregnancy, breastfeeding, and renal impairment) or transcranial magnetic stimulation (TMS) (seizure, pacemaker, or ferromagnetic material in the head area) were also excluded. Eligible patients received oral fampridine 10 mg twice daily for two consecutive weeks.

### 2.3. Clinical and Neurophysiological Assessments

Assessments were performed at baseline (T0) and 14 days after the commencement of fampridine (T1). The physicians performing the clinical assessment were blinded to the cortical excitability results and vice versa. 

Clinical assessment was performed by N.R., S.E.N. and M.M.S.. At baseline (T0), a detailed medical history was obtained with particular emphasis on key MS related elements: Onset and type of MS, number and dates of clinical relapses, prior disease modifying drugs, comorbidities, and current medications. At the follow-up visit (T1), the interview was focused on drug side effects and subjective improvement. Particular attention was paid to the occurrence of any new symptom or any worsening of a pre-existing deficit that could be indicative of a new relapse. A thorough neurological examination and an assessment of disability status using the Kurtzke Expanded Disability Status Scale (EDSS) were done at baseline (T0) [14]. Cortical excitability assessment and a timed 25-foot walk test (T25wt) [15] were done during both visits (T0 and T1). 

Decrease in T25wt was calculated as follows: ΔT25wt = T25wt at T0 − T25wt at T1. Then, the percentage of improvement was calculated as follows: % of improvement = $\frac{\Delta T25wt}{T25wtatT0}$ × 100. 

Patients who showed a percentage of improvement greater than 20% were considered responders. This threshold was based on the finding that a 20% improvement in walking time is considered clinically meaningful for patients with MS [16]. 

Cortical excitability studies were performed by R.A. and included the resting motor threshold (rMT), short-interval cortical inhibition (SICI), intracortical facilitation (ICF), and cortical silent period (CSP). MEPs were recorded from the first dorsal interosseous muscle (FDI) using pre-gelled disposable surface electrodes in a tendon-belly montage (ref 019-400400, Natus, Pleasanton, CA, USA). Recording was done on the side affected least by MS or on the left side if both sides were normal or equally affected. 

MEPs were filtered (20 Hz to 2 KHz), amplified, and stored for off-line analyses (Nicolet EDX, Natus, Pleasanton, CA, USA). Patients were seated in a comfortable chair with the arms at rest. Baseline muscle activity was continuously monitored to ascertain complete muscle relaxation (except for CSP measurement). A tight-fitting cap was placed on the patient’s head to help mark the coil position. Magnetic stimulation was delivered using an eight-shaped coil with an inner diameter of 70 mm (M200^2^ D70 double coil 3190-00, Magstim Co, Carmarthenshire, U.K.) placed tangentially to the scalp (handle pointing backwards) and connected to a Magstim Bistim^2^ stimulator (Magstim Co, Carmarthenshire, U.K.). 

The motor hot spot was determined by scanning the scalp for the coil position associated with the largest MEPs. This position was marked on the cap and used for all subsequent excitability measurements. It was determined in a fully relaxed FDI muscle and defined as the lowest stimulus intensity required to produce MEPs larger than 50 µV (peak-to-peak) in 5 out of 10 consecutive trials [17]. 

A paired stimulus paradigm was used to test SICI using interstimulus intervals (ISIs) of 2 and 4 ms (SICI2 and SICI4 respectively) [18]. ICF was tested using ISIs of 10 and 15 (ICF10 and ICF15, respectively) [18]. The conditioning stimulus was delivered at 80% of rMT and the test stimulus was delivered at 120% of rMT. Eight trials were performed for each condition. The average of eight trials of unconditioned TMS pulses delivered at 120% of rMT was used as control. The amplitudes of conditioned MEPs were expressed as the percentage of the mean unconditioned MEP amplitude. The mean degree of inhibition and facilitation from all tested ISIs were retained for analysis.

CSP was defined as the duration of electromyogram (EMG) activity interruption following a single TMS pulse delivered at 140% of the rMT. The degree of FDI activation was controlled by visual feedback. Five trials were performed and averaged. The minimal CSP duration was measured from the end of the MEP until the first reoccurrence of EMG activity on highly magnified traces [19]. 

This study was sponsored by an investigator-initiated trial grant from Biogen, who reviewed the protocol. Biogen did not participate in patient recruitment or study implementation, had no access to the data, and did not participate in the statistical analysis. 

### 2.4. Statistical Analysis

The analysis was conducted using the R statistical program 3.5.3 (R Foundation for statistical computing, Vienna, Austria). Descriptive statistics are presented as mean ± standard deviation (SD) and median (interquartile range) for continuous variables. 

The Wilcoxon rank sum paired test was used to assess changes in cortical excitability parameters (comparison between measurements at T0 and T1), and Pearson correlation coefficient was used to analyze the relationship between ΔT25wt and baseline cortical excitability measures (rMT, MEPs amplitude, SICI, ICF, CSP) and between ΔT25wt and EDSS scores. The relationship between EDSS and baseline excitability measures was also examined. A *p*-value <0.05 was considered significant. A classification model using the C5.0 decision tree algorithm (Quinlan R (1993). C4.5: Programs for Machine Learning. Morgan Kaufmann Publishers [20] was used to classify the samples as fampridine responders and non-responders based on their rMT value. As stated previously, patients were considered responders if they had at least a 20% reduction in their T25wt at the follow-up visit (T1), and non-responders otherwise. A simple model was built using the formula “responder status ~ rMT” while keeping the default values for all the remaining parameters in the model. The C5.0 decision tree algorithm relies on the maximum information gain to identify the rMT cutoff value that best splits the samples as responders and non-responders to estimate a threshold value that discriminates between fampridine responders and non-responders. The model was built using the C5.0 from the C50 package in R.

## 3. Results

### 3.1. Clinical and Sociodemographic Data

A total of 20 patients (11 male) completed the study (Table 1). They had a mean age of 49.75 ± 11.36 (median interquartile range (IQR): 50.00 (18.00); overall range: 25–65) years. Thirteen had relapsing remitting, 6 had secondary progressive, and one had primary progressive MS. Mean disease duration was 13.75 ± 8.17 years (median (IQR): 12.50 [10]; overall range: 3–39). Their mean EDSS score was 4.70 ± 1.31 (median (IQR): 4.00 (1.88); overall range 2.5–7). Nineteen patients were on disease modifying treatment: 3 on interferon beta 1a, 12 on fingolimod, 3 on natalizumab, and 1 on dimethyl fumarate. 

### 3.2. Clinical Response to Fampridine 

Significant improvement in T25wt was found following fampridine (21.5 ± 5.1 versus 16.64 ± 1.40 , *p* < 0.001). Mean ΔT25wt was 4.86 ± (range 0.04–17.62) seconds (s). Eleven patients (55%) were responders to fampridine, as defined previously. 

The treatment was well tolerated, and no serious adverse effects were reported. Thirteen patients (65%) reported minor side effects, mostly nausea, abdominal pain, lumbar/cervical pain, and dizziness. There were no instances of patient withdrawal owing to treatment side effects.

### 3.3. Effects of Fampridine on Cortical Excitability Measures

As depicted in Table 2, the follow-up visit (T1) showed a statistically significant enhancement of ICF15 (139.17 ± 110.98% versus 241.69 ± 131.46%, *p* = 0.01). ICF10 also tended to increase, but this effect did not reach statistical significance (161.58 ± 154.12% versus 242.94 ± 125.79%, *p* = 0.06) (Table 2). No significant changes were observed for the rMT (60.95 ± 12.47% versus 62.45 ± 15.20%, *p* = 0.81), SICI2 (53.08 ± 68.24% versus 73.62 ± 69.95%, *p* = 0.11), SICI4 (88.18 ± 107.10% versus 121.51 ± 88.48%, *p* = 0.15), or CSP (132.15 ± 55.67 ms versus 138.60 ± 51.90 ms, *p* = 0.92). 

### 3.4. Relationship Between EDSS and Baseline Excitability Measures

At baseline (T0), a positive correlation was found between EDSS and rMT (*r* = 0.57; *p* < 0.01) (Table 3). Conversely, no statistically significant correlation was found between EDSS and any of the other cortical excitability measures. 

### 3.5. Relationships Between Improvement of T25wt (ΔT25wt) and Each of EDSS and Baseline Cortical Excitability Measures 

A statistically significant positive correlation was found between baseline rMT and improvement of walking speed at the follow-up visit (*r* = 0.57, *p* = 0.01). No correlation was found between ΔT25wt and other baseline excitability parameters. 

A positive correlation was also found between ΔT25wt and EDSS. (*r* = 0.75; *p* < 0.01) (Table 3).

### 3.6. Predictors of a Favorable Response to Fampridine

The predictive value of baseline rMT in terms of response to fampridine was studied. The threshold to predict good response to fampridine was found to be 52 (with 14 patients with rMT above 52 were found to be good responders to the treatment). Mean ± SD of rMT in responder and non-responder groups were 64.10 ± 11.99 and 57.11 ± 12.62, respectively. The classification of responders and non-responders had an accuracy of 75% (specificity: 83.3% (5/6) and sensitivity 71.4% (10/14)). 

## 4. Discussion

Three interesting findings emerged from our study. First, higher rMT at baseline was predictive of a favorable response to fampridine, translating clinically into improved gait speed. Conversely, the other excitability parameters were not found to have any predictive value. From a clinical standpoint, patients with higher EDSS showed enhanced drug effects compared to those with lower EDSS. At a more mechanistic level, fampridine was found to induce a significant increase in intracortical excitatory mechanisms as revealed by paired-pulse TMS, with no significant effect on the other excitability parameters. 

The positive impact of fampridine on gait speed is now well established, but only 40% of patients are expected to experience a clinically meaningful improvement [15,21]. Proper selection of patients most likely to experience clinical benefits from fampridine has been the subject of rare dedicated studies [6,7,8]. It has been suggested that patients with more disability at baseline have the best outcome. This seems logical, since advanced ambulatory impairment in MS is associated with a higher amount of axonal demyelination within the neural locomotor networks, providing more targets for fampridine to reinforce gait function [6,8]. Filli and collaborates tested the predictive value of a set of demographic and clinical criteria including T25wt, walking endurance 6-min walk test (6MWT), and the 12-item multiple sclerosis walking scale (MSWS-12) [7]. Their findings suggest that walking function at baseline (6MWT and T25wt) accurately predicts the responder status. EDSS only weakly correlated with the outcome. In this study, reduced walking endurance as measured by the 6MWT was the best predictor of a good outcome with an accuracy of 80% [7]. In comparison, we found that EDSS correlated with improvement in T25wt; however, we did not test walking endurance, since we included patients with advanced gait impairment who were unable to complete the 6MWT. 

At the physiological level, high rMT was found to be correlated with high EDSS score, and seemed to be a good predictor of outcome. rMT is a global measurement of excitability and membrane properties of cortical pyramidal cells [22,23]. It can be regarded as the electrophysiological signature of clinical impairment in MS patients. Higher rMT was found to correlate with clinical relapses [24], disease progression [25], secondary progressive disease [10,26], and the presence of fatigue [11]. Another neurophysiological measure of potential predictive value is prolonged motor central conduction [6,8]. The latter parameter was not evaluated in our study. 

Studies of cortical excitability, a surrogate measure of ion channel and synaptic functions, have repeatedly shown significant excitability changes in patients with MS [10,11,12]. In the earlier stages of relapsing remitting MS, this state of altered excitability can be improved by immunomodulatory therapy [27]. As the disease progresses and the degenerative process takes place, excitability changes could become permanent [10,26]. At this stage, drugs having a more direct effect on cortical excitability are more likely to reverse these changes than those with immunomodulatory effects. Our study clearly demonstrates a cortico-modulatory effect of fampridine, a rather predictable finding given its putative mechanism of action. Cortical excitability measures are dependent on networks of interneurons (both excitatory and inhibitory) and their synaptic interaction with each other. SICI reflects the recruitment of inhibitory pathways with GABAergic mediation (GABA-A receptors) and ICF reflects the recruitment of excitatory pathways with glutamatergic mediation. Healthy excitatory and inhibitory systems are essential for proper functioning of brain circuits. Fampridine was found to increase ICF in our study but had no impact on the other excitability parameters. Such findings would indicate an increase in facilitatory intracortical mechanisms aiming to improve the clinical outcomes, as reflected by an increase in walking speed [28,29].

Our study has many shortcomings, including an open label design, small sample size, and short follow-up period. Another shortcoming is that cortical excitability changes were correlated to changes in walking speed, but not other cortical functions. Since cortical excitability studies are classically measured in the hand, correlations with hand function (i.e., nine-hole pegboard test) would have been more appropriate, especially as fampridine has been shown to have a positive impact on hand function [30]. Alternatively, we could have chosen a lower extremity muscle (rather than the FDI) to measure lower extremity cortical excitability. This choice of the FDI was based on the many challenges associated with measuring cortical excitability in lower extremities, such as the deeper location of the lower extremity motor areas (making them significantly more difficult to activate), the difficulty of targeting individual lower extremity muscles, and the paucity of studies investigating the reliability and reproducibility of lower extremity excitability studies. Yet another limitation is the short follow-up period, which was limited to 2 weeks. It has been demonstrated that walking speed can continue to improve up to 4 weeks after fampridine initiation [15,21]. As such, a longer period of assessment might have been required to more reliably define the responder status. These shortcomings were inevitable, given the observational nature of the study. Indeed, the inclusion criteria and follow-up period were dictated by our national guidelines for selecting candidates for fampridine therapy. As such, inclusion was restricted to patients with gait impairment, most of which had little or no baseline abnormalities in the hands, and the follow-up period was limited to 2 weeks. Our study could also be criticized for including 10 patients on fingolimod, which was found to reduce ICF [4]. Since no changes in disease modifying and symptomatic treatments were allowed during the period extending from 6 months prior to inclusion to the end of the follow-up, this drug would have mostly affected the baseline recordings rather than the changes observed after fampridine administration. Patients receiving high-dose intravenous steroids, on the other hand, were excluded from the study, given its significant impact on cortical excitability (reduction of SICI and enhancement ICF) [9]. Lastly, it is important to mention the limitations of EDSS as a disability measure in MS. The latter has well documented weaknesses in reliability and sensitivity to change [31].

## 5. Conclusions

In conclusion, our work suggests that fampridine has a significant modulatory effect on the cerebral cortex, demonstrated by an increase in excitatory intracortical mechanisms as unraveled by paired-pulse TMS paradigms. rMT could be useful in selecting patients more likely to experience a favorable response to fampridine. Larger studies with more reliable outcomes (i.e., hand function) are needed to better define the cortical excitability parameters that best discriminate between potential responders and non-responders.

## Figures and Tables

**Table 1:** Baseline characteristics of subjects.

Subjects	Age	Sex	Disease Duration	EDSS	MS Type	Present Medication
1	53	F	7 years	5	RRMS	Fingolimod
2	46	M	11 years	4	SPMS	Fingolimod
3	25	M	5 years	4	RRMS	Fingolimod
4	49	F	10 years	7	RRMS	Natalizumab
5	62	M	18 years	5	RRMS	Fingolimod
6	62	F	22 years	5.5	SPMS	Interferon β 1a
7	42	F	17 years	4	SPMS	Natalizumab
8	47	F	8 years	5.5	RRMS	Fingolimod
9	58	M	19 years	4	SPMS	Fingolimod
10	58	F	12 years	7	SPMS	Fingolimod
11	62	M	11 years	4	PPMS	None
12	51	F	13 years	6	SPMS	Dimethyl Fumarate
13	65	M	18 years	6	RRMS	Fingolimod
14	50	F	18 years	6.5	RRMS	Fingolimod
15	35	M	3 years	3.5	RRMS	Fingolimod
16	32	M	3 years	3	RRMS	Natalizumab
17	50	M	18 years	4	RRMS	Interferon β 1a
18	64	M	39 years	3.5	RRMS	Interferon β 1a
19	36	M	15 years	4	RRMS	Fingolimod
20	48	F	8 years	2.5	RRMS	Fingolimod

EDSS: Expanded Disability Status Scale; F: female; M: male; MS, Multiple sclerosis; PPMS: Primary progressive multiple sclerosis; RRMS: Relapsing remitting multiple sclerosis; SPMS: Secondary progressive multiple sclerosis.

**Table 2:** Observed measures of excitability parameters and timed 25-foot walk test (T25wt) at baseline in seconds (s) and after fampridine treatment.

Parameter	Baseline Mean ± SD Median (IQR)	After Fampridine Mean ± SD Median (IQR)	Wilcoxon Paired-Test *p*-Value
rMT	60.95 ± 12.47 57.50 (19.75)	62.45 ± 15.20 58.50 (30.50)	0.81
SICI2	53.08 ± 68.24 31.95 (53.22)	73.62 ± 69.95 50.00 (70.94)	0.11
SICI4	88.18 ± 107.10 47.05 (52.77)	121.51 ± 88.48 107.90 (150.17)	0.15
ICF10	161.58 ± 154.12 134.61 (172.06)	242.94 ± 125.79 217.67 (210.46)	0.06
ICF15	139.17 ± 110.98 114.75 (139.63)	241.69 ± 131.46 235.00 (182.49)	**0.01**
CSP	132.15 ± 55.67 119.00 (54.25)	138.60 ± 51.90 147.50 (71.25)	0.92
T25wt	21.5 ± 5.1	16.64 ± 1.40	**<0.001**

The short intracortical inhibition at interstimulus intervals (ISIs) of 2 and 4 ms (SICI2, SICI4), intracortical facilitation at ISIs of 10 and 15 ms (ICF10, ICF15) are expressed as the percentage of the unconditioned motor evoked potential (MEP) amplitude. The cortical silent period (CSP) is expressed in ms. The resting motor threshold (rMT) is expressed as percentage of the maximal stimulator output. *p*-values in bold reflect significant difference (*p* < 0.05). IQR, interquartile range.

**Table 3:** Correlation between the Expanded Disability Status Scale (EDSS) and baseline measures of resting motor threshold (rMT), short-interval intracortical inhibition at interstimulus intervals (ISIs) of 2 and 4 ms (SICI2 and SICI4, respectively), intracortical facilitation at ISIs of 10 and 15 (ICF10 and ICF15, respectively), cortical silent period (CSP), and timed 25-foot walking test (T25wt).

	Variables	Correlation	*p*-Value
EDSS	rMT	0.57	<0.01
	SICI2	−0.24	0.30
	SICI4	−0.27	0.24
	ICF10	−0.18	0.42
	ICF15	0.007	0.97
	CSP	−0.08	0.71
	ΔT25wt	0.75	<0.01
